# Defoliation Change of European Beech (*Fagus sylvatica* L.) Depends on Previous Year Drought

**DOI:** 10.3390/plants11060730

**Published:** 2022-03-09

**Authors:** Mladen Ognjenović, Ivan Seletković, Nenad Potočić, Mia Marušić, Melita Perčec Tadić, Mathieu Jonard, Pasi Rautio, Volkmar Timmermann, Lucija Lovreškov, Damir Ugarković

**Affiliations:** 1 Division for Forest Ecology, Croatian Forest Research Institute, 10450 Jastrebarsko, Croatia; mladeno@sumins.hr (M.O.); ivans@sumins.hr (I.S.); miam@sumins.hr (M.M.); lucijal@sumins.hr (L.L.); 2 Meteorological and Hydrological Service, 10000 Zagreb, Croatia; melita.percec.tadic@cirus.dhz.hr; 3 Earth and Life Institute, Université Catholique de Louvain, 1348 Louvain-La-Neuve, Belgium; mathieu.jonard@uclouvain.be; 4 Natural Resources Institute Finland, 00790 Helsinki, Finland; pasi.rautio@luke.fi; 5 Norwegian Institute of Bioeconomy Research, 1433 Ås, Norway; volkmar.timmermann@nibio.no; 6 Faculty of Forestry and Wood Technology, University of Zagreb, 10000 Zagreb, Croatia; dugarkovic@sumfak.hr

**Keywords:** defoliation, monitoring, tree vitality, drought, climate change

## Abstract

European beech (*Fagus sylvatica* L.) forests provide multiple essential ecosystem goods and services. The projected climatic conditions for the current century will significantly affect the vitality of European beech. The expected impact of climate change on forest ecosystems will be potentially stronger in southeast Europe than on the rest of the continent. Therefore, our aim was to use the long-term monitoring data of crown vitality indicators in Croatia to identify long-term trends, and to investigate the influence of current and previous year climate conditions and available site factors using defoliation (DEF) and defoliation change (ΔDEF) as response variables. The results reveal an increasing trend of DEF during the study period from 1996 to 2017. In contrast, no significant trend in annual ΔDEF was observed. The applied linear mixed effects models indicate a very strong influence of previous year drought on ΔDEF, while climate conditions have a weak or insignificant effect on DEF. The results suggest that site factors explain 25 to 30% DEF variance, while similar values of conditional and marginal *R*^2^ show a uniform influence of drought on ΔDEF. These results suggest that DEF represents the accumulated impact of location-specific stressful environmental conditions on tree vitality, while ΔDEF reflects intense stress and represents the current or recent status of tree vitality that could be more appropriate for analysing the effect of climate conditions on forest trees.

## 1. Introduction

Climate conditions influence the structure and function of forest ecosystems, and play an essential role in forest health [1,2]. Global warming has indisputably caused climate change, which is a significant threat to forest ecosystems [3]. The effects of climate change are generally expected to reduce tree growth and survival, predispose forests to disturbances, and ultimately change forest structure and composition at the landscape scale [4]. Therefore, there is an increasing concern in Europe over the sustainability of forest ecosystems under climate change [5].

Although vitality is a theoretical concept, it can be defined as the ability of a tree to assimilate, to survive stress, to react to changing conditions, and to reproduce [6]. As vitality cannot be measured directly, various indicators can be used to describe it [7]. Crown defoliation is a commonly used tree vitality indicator [8,9,10], which can be obtained cost-effectively and relatively quickly in field surveys [11]. Defoliation is defined as leaf loss in the assessable crown, as compared to a reference tree, and is observed regardless of the cause of foliage loss [12]. Landmann [13] states that defoliation is an indicator of acute stress and subsequent recovery of forest ecosystems. However, defoliation has been criticized due to the subjectivity of the assessment, as well as it being a non-specific indicator affected by several biotic and abiotic factors [14,15,16]. To ensure data quality, training courses and repeated control assessments are regularly carried out on a national [17,18,19] and international level [20].

European beech (*Fagus sylvatica* L.) is a dominant broadleaved tree species in European forests that forms forest communities over a broad range of habitat conditions [21]. These forests provide multiple ecosystem goods and services [22]. Despite being adapted to a wide range of environmental conditions, the projected effects of climate change, particularly drought, will significantly affect the vitality of European beech [23,24].

Southeast Europe represents one of the most vulnerable regions with expected intensification of severity and duration of droughts and heat waves. As the impacts of climate change on forests in southeast Europe will be potentially stronger and faster than on the rest of the continent [25,26], this region is an ideal model for studying the impacts of changing climatic conditions. A trend of decreasing precipitation and increasing temperatures has already been observed in Croatia [27,28]. In the decade 2001–2010 alone, four drought events occurred, while only 13 took place between 1961 and 2010 [29]. In the future, the climate in Croatia is expected to be hotter and drier, with considerable impacts to be expected for the forest ecosystems. Consequently, continuous long-term forest monitoring is crucial in order to measure and assess these impacts and their consequences on ecosystem functioning.

In Europe, the International Co-operative Program on Assessment and Monitoring of Air Pollution Effects on Forests (ICP Forests) is the most comprehensive European program for the large-scale assessment of forest ecosystem health [30]. The defoliation data obtained from the ICP Forests monitoring network have led to the publication of numerous studies of climate influence in several European countries, such as Switzerland [31], Germany [32], France [33], and Spain [34]. Depending on the investigated region, different climate parameters were found to have a negative impact on crown defoliation. Studies of this kind in Croatia have previously only been regional, which has limited the applicability of results [35,36]. A recent pilot study found a pronounced lag effect of both temperature and precipitation on beech defoliation [37]. Based on these studies, we hypothesize that previous and current year droughts as a consequence of high temperatures and low precipitation contribute the most to beech defoliation across Croatia. Given the ecological and economic importance of beech, it is necessary to understand the impact of climate change on beech defoliation. Therefore, by using the ICP Forests monitoring network in Croatia, our aim was to (i) identify European beech long-term defoliation trends, and to (ii) investigate the influence of current and previous year climate conditions and various site factors on beech defoliation.

## 2. Results

### 2.1. Temporal Trends in Tree Vitality

**Figure 1:**
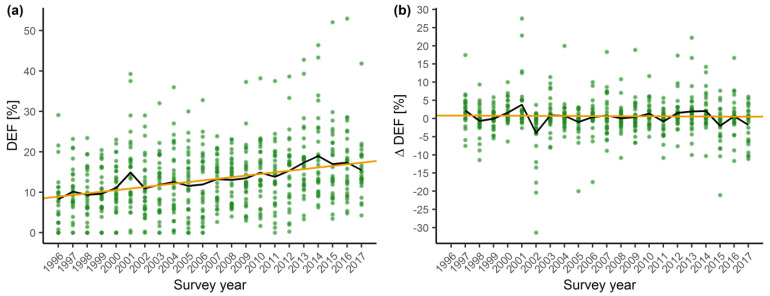
Overall trend of crown vitality parameters from 1996–2017. (**a**) Defoliation trend of European beech (Tau = 0.78, Sen’s slope = 0.39, *p* < 0.001, orange line) and annual overall mean defoliation (DEF*_i_*, black line). (**b**) Defoliation change trend of European beech (Tau = −0.05, Sen’s slope = −0.01, *p* = 0.73, orange line) and annual overall change in mean defoliation (ΔDEF*_i_*, black line). Points represent annual plot mean values.

There was a significant trend of increasing mean defoliation (DEF) by 0.39% annually over the study period (Figure 1). Annual overall mean defoliation values peaked in 2001 and 2014. The latter year is marked by the highest observed overall mean defoliation of 18.4%. In contrast, no significant trend in annual defoliation change (ΔDEF) was observed. Time series of annual defoliation change values exhibit stationarity (Dickey–Fuller = −5.81, *p* < 0.01), and generally stay close to the neutral trend line.

Plot-scale trend analysis of DEF showed that 53% of plots have a significant and increasing trend of defoliation at an annual rate ranging from 0.25% to 1.29%. On the remaining 47% of plots, we did not observe any significant trend. The results of the q-statistics and Morans’ I spatial autocorrelation coefficient did not indicate spatial stratification of plot-scale DEF trends (data not shown). We did not detect any significant ΔDEF trend at the plot-scale. Therefore, the subsequent spatial stratification tests were not conducted.

### 2.2. Influence of Environmental Conditions on Tree Vitality

During the selection procedure for DEF and ΔDEF models, additional variables were considered: stand age, site factors (altitude and orientation), and soil properties (soil pH, total nitrogen content in the soil, content of available phosphorus and available potassium in the soil) were tested. However, only altitude showed a significant impact. Although the influence of stand age was not significant, it was retained in the selection process due to improved model performance and the reported influence of stand age on defoliation in other studies [38,39].

The two linear mixed effects models (LMM) used to assess the impact of climatic variables on DEF revealed different influences of current and previous year SPEI (Standardized Precipitation Evapotranspiration Index). Current year SPEI is positively correlated with defoliation, while previous year SPEI has a negative effect. Although significant, this divergent effect of drought should be regarded with caution, since the estimated effects are weak (Table 1). Both the current and previous year temperature and precipitation did not have significant effects on defoliation. Plots located on higher altitudes had significantly higher mean defoliation. The annual increase in defoliation estimated in the LMM models is very similar to the positive defoliation trend assessed by the Mann–Kendall test (Figure 1). The marginal coefficient of determination (*R*^2^) was lower than the conditional *R*^2^ in both models, which suggests that the model random effects i.e., plot location, accounts for a high proportion of the explained DEF variance (Table 2).

**Figure 2:**
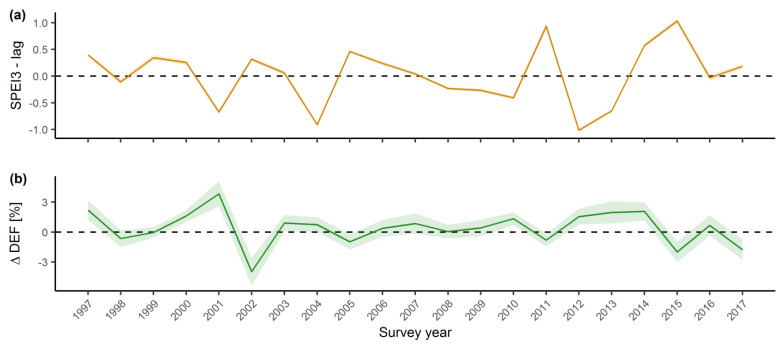
Inverse relationship between previous year SPEI (Standardized Precipitation Evapotranspiration Index) and defoliation change (ΔDEF*_i_*) in Croatia from 1996–2017. (**a**) Previous year mean SPEI (orange line) and standard error (orange area), calculated on a time scale of three months. (**b**) Annual overall defoliation change (ΔDEF*_i_*) mean (green line) and standard error (green area).

Defoliation change LMM indicates a very strong and significant negative influence of previous year SPEI (Table 3). Positive defoliation changes were observed in years preceded by low SPEI, while negative defoliation changes were associated with high SPEI the previous year. This inverse relationship where the increase of defoliation is preceded by drought was most notable in 2001 (Figure 2). Other climate variables, including current year SPEI, did not have a significant effect and neither did stand age or elevation. Equal values of conditional and marginal *R*^2^ suggest a uniform effect of previous year drought on European beech ΔDEF (Table 2). As expected, the models did not reveal a significant trend in the change of defoliation.

## 3. Discussion

### 3.1. Temporal Trends in Crown Vitality

Defoliation is widely accepted as a proxy indicator of tree vitality and forest health, able to provide useful information on its status and trends. Long-term defoliation data series are an important asset to explore the changes in forest ecosystem health across Europe over the past 30 years [8]. This study revealed a statistically significant trend of increasing defoliation of European beech in Croatia over time. The distinctive impulses of increasing mean defoliation in 2001 and from 2012 to 2014 (Figure 1) are preceded by the dry 2000 and the extreme drought period recorded in 2011/2012 (Figure S1) [40]. The severe drought recorded in 2003 did not result in an increase in mean defoliation in the following years, which is consistent with our previous findings [37]. Sudden increases in mean defoliation preceded by years or periods of pronounced precipitation deficit were also recorded in studies conducted in France [33] and the Iberian Peninsula [41]. A significant, but weaker trend of increasing beech defoliation was also observed at a European level [42]. A study comparing the general defoliation trends between geographical regions found that southern Europe, including Croatia, has a more pronounced trend of increasing defoliation compared to central and northern Europe [41].

Plot-scale analysis of defoliation revealed a statistically significant increase on 53% of plots in the 1996 to 2017 period. A study applying a similar plot-scale approach in France found that as many as 70% of beech plots showed an increasing defoliation trend from 1996 to 2009 [33]. Our results also suggest that there is no spatial grouping of the defoliation trend, which is in line with other studies [9,31]. Obviously, the trend of defoliation is not influenced by geographical position, but rather by specific environmental conditions present on a particular plot.

Defoliation change (ΔDEF), defined as the difference between the defoliation assessed in the current and previous year has so far been used in only a few studies [31,43]. Unfortunately, detailed results from these studies were not provided, and therefore a straightforward comparison with our results was not possible. While differences in assessment of absolute defoliation values can be expected due to national adjustments of the methods, the differences in assessing the relative change of defoliation from year to year should be negligible, and could potentially reduce the influence of possible subjectivity of the assessment [43]. Additionally, the absence of serial correlation of defoliation change, i.e., its stationarity, enables easier development of impact models compared to using defoliation data.

### 3.2. Influence of Environmental Conditions on Tree Vitality

A key task at the European level is to study the impact of climate change on crown defoliation and, consequently, on forest health [44], taking into consideration a wide range of natural and anthropogenic environmental factors [45].

The established difference in marginal and conditional *R*^2^ in crown defoliation models suggests that site factors explain 25 to 30% variance of European beech defoliation over time. A high influence of specific site attributes on defoliation was also found in other studies, e.g., lower defoliation values were observed at higher nitrogen supply levels and higher pH levels [46,47]. However, soil properties did not show a significant impact during the model selection process in our study, which is in line with several studies that did not confirm the importance of soil properties for European beech defoliation [10,43]. It is possible that the applied approach to soil sampling and analysis does not provide a sufficient level of detail to detect the significance of soil variables, given that soil properties have nevertheless been shown as significant factors in some studies of European beech [48] and Norway spruce defoliation [49]. On the other hand, effects from environmental factors that change on a long-term scale, like soil properties, will not likely be detected through annual variation of defoliation [46].

Stand age was identified as a significant predictor in different approaches to modelling defoliation [39,40,45,50]. However, the established relationship between defoliation and stand age may, in many cases, represent an interaction between various stress factors and age [4]. The absence of a significant impact of stand age on European beech defoliation in Croatia (Table 1 and Table 3) can be explained by a relatively small number of plots in our sample, where stands are older than 80 years (Figure S2), while the abovementioned studies were not limited by irregular age distribution.

Numerous studies have observed a significant effect of drought on increasing defoliation [32,38,39,42,45], and increased leaf loss following spring and summer heat waves was recorded both in European [50,51] and North American forests [52]. In contrast to the clear influence of spring and summer temperatures on European beech defoliation found in Spain [34], the results of this study indicate a very weak influence of all examined temperature variables, which is consistent with results of a beech study conducted in Germany [46]. Furthermore, we did not find a significant influence of precipitation on defoliation. This is contrary to the results of a French study, where precipitation and precipitation deficit correlated with defoliation [33]. While we could not detect any effect of air temperature and precipitation when considered independently, these climate variables showed to have a clear impact on defoliation change when combined in the SPEI drought index.

The basic mechanism for regulating water loss in dry conditions is stomatal closure in plants [53]. Under conditions of increased water deficit, plants also respond by increasing water use efficiency [54], reduced growth [55], and conservative mechanisms such as limiting their photosynthetic activity [56]. Due to drought, plants can adapt their morphological structure by increasing the carbon allocation to the root system [57], reducing their leaf size [58], decreasing leaf area index [59], and ultimately shedding leaves [10]. During long lasting drought events, stomatal closure can significantly reduce carbon fixation by trees as well as their carbon reserves, which weakens trees and makes them more vulnerable to biotic and abiotic stresses. In extreme cases, this can lead to mortality by carbon starvation [60]. Severe drought during the year of bud formation, in our study indicated by low previous year SPEI and its impact on ΔDEF, decreases the number of new leaves formed in the bud thus influencing the number of leaves, leaf surface area, and twig extension in the following year [51]. In *Fagus* species, all leaves are pre-formed in winter buds [61,62] during late summer and early autumn [59]. Hydraulic failure may also occur during severe droughts leading to twig and leaf abscission, which can be seen as a drastic adaptation strategy to reduce evapotranspiration [63]. This effect is visible from the values of defoliation rising in the period from 2011 until 2014 (Figure 1), which coincides with low SPEI for the years 2011 and 2012 (Figure 2).

Equal values of conditional and marginal *R*^2^ and the low value of ICC (intraclass correlation coefficient) suggests a uniform influence of previous year drought on the European beech ΔDEF throughout Croatia. On the other hand, the increase in the marginal *R*^2^ of defoliation models after the inclusion of climatic variables was slight, and the influence of all observed climatic variables on DEF was weak. This indicates that defoliation is influenced by site-specific environmental or stand factors that have not been identified in this study. Mean beech defoliation shows fluctuations that coincide with the occurrence of common to abundant fructification [48,64]. However, we were not able to include fruiting as a factor due to the lack of data. The lack of a clear ΔDEF trend, as well as the pronounced impact of drought in the previous year, may indicate that this response variable reflects intense stress, while the positive DEF trend represents the accumulated impact of location-specific stressful environmental conditions on tree vitality. Since defoliation change shows the current or recent status of tree vitality, while defoliation is an integrated indicator resulting from cumulated biotic and abiotic pressures on tree vitality over many years, defoliation change could be a more appropriate indicator for analysing the effect of recent climate conditions on tree vitality.

Increasing temperatures may lead to drought thus affecting forest vitality in the region. Forest monitoring activities in southeast Europe should be intensified to determine the unknown site-specific environmental and/or stand factors that may explain a part of the variance in the present data. This could help develop adequate and locally applicable mitigation strategies to secure the future of beech forests in the region.

## 4. Materials and Methods

### 4.1. Study Area and Plot Selection

**Figure 3:**
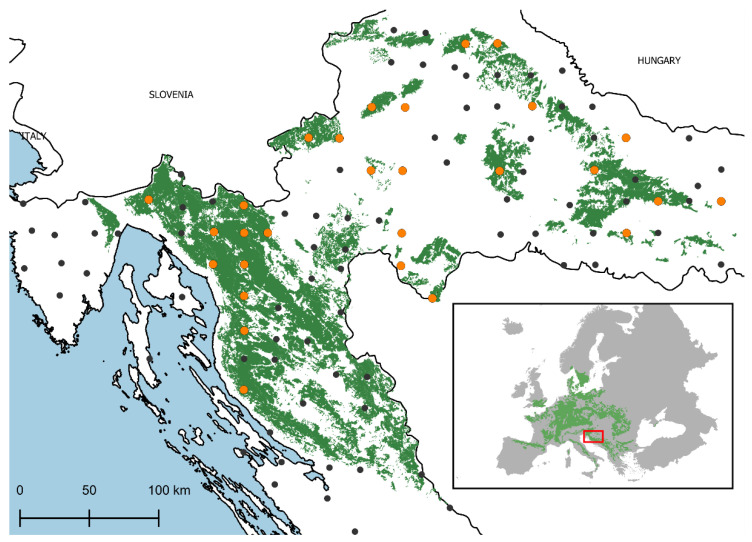
Location of research plots in the context of the distribution of European beech in Europe [66]. Selected Level I ICP Forests monitoring plots (orange dots); remaining Level I plots in Croatian (black dots); distribution of European beech forests in Croatia [67] (green polygon).

The ICP Forests Level I monitoring plots in Croatia are established on intersections of a 16 × 16 km grid that contain forest cover. These plots do not have a fixed area; rather, 24 trees are chosen for defoliation assessments using a cross-cluster system with six trees in each cluster [65]. Only plots with a minimum of five European beech trees were selected to ensure that European beech was significantly represented in the mixture of tree species. To ensure defoliation data consistency over the investigated period from 1996 to 2017, defoliation assessment on selected plots had to have been carried out for at least 80% of the investigated period. This resulted in the selection of 28 research plots (Figure 3). In addition to defoliation, the ICP Forests database contains information on several site factors (Table S1).

### 4.2. Defoliation Assessment and Crown Vitality Indicators

Defoliation of European beech trees on the selected plots was assessed annually between mid-July and mid-August from 1996 to 2017, in 5% classes from 0 to 100%, according to the ICP Forests Manual [12]. Assessments of tree crowns was performed in comparison with the absolute reference tree. For this study, two crown vitality indicators were calculated and used as response variables: (i) the mean current year crown defoliation DEF*_i_* on plot *i*, and (ii) the change in the mean current year crown defoliation on plot *i* compared to the previous year assessment ΔDEF*_i_*. Mean values of crown vitality parameters at the plot level were used, since the values of all predictor variables could only be obtained at the plot level. Additionally, comparison of defoliation variability within and between plots showed that it was lower within plots than between plots.

### 4.3. Soil Sampling and Analysis

Soil sampling was performed during the summer of 2019 on five points located within each of the research plots. One point was located within each of the four groups of trees that are assessed for defoliation, and an additional fifth point was located in the centre of each research plot. Soil samples were taken with a pedological drill from a depth of 0–10 cm, 10–20 cm, 20–40 cm, and 40–80 cm. Collected samples were pooled according to the sampling depth. Soil chemical parameters were analysed according to standard protocols and methods (Table S2).

### 4.4. Climate Data

Climate monitoring stations are generally situated at considerable distances from the research plots. Therefore, the data they provide are not always representative of the research locations. To overcome this, we used gridded data produced by regression kriging (RK), which is a hybrid method of interpolation carried out in four steps [68]. The method was validated with leave-one-out cross-validation, while the root mean square error (RMSE) was calculated between observed and interpolated values. Mean RMSEs are for mean monthly temperature from 0.5 °C to 0.9 °C, for minimum temperature from 1.1 °C to 1.5 °C, for maximum temperature from 0.7 °C to 1.1 °C, and for precipitation from 18 to 30 mm, averaged by months.

Mean monthly temperature (T), minimum (Tmin) and maximum (Tmax) monthly temperature, and monthly sum of precipitation (P) from the gridded dataset on 1 km spatial resolution for Croatia [69] were used to calculate yearly values, as well as the Palmer Drought Severity Index (scPDSI) [70] and Standardized Precipitation Evapotranspiration Index (SPEI) [71]. Lower values of scPDSI and SPEI indicate a stronger drought intensity while higher values indicate a higher degree of humidity. SPEI was calculated on a time scale of 3, 6, and 12 months.

### 4.5. Data Analysis

The trend of defoliation and defoliation change was estimated according to Sen’s slope [72], while the significance of a trend was tested by the Mann–Kendall test [73,74] with a significance level of *p* ≤ 0.05. Both methods are suitable for data with asymmetric distribution and in this case are significantly more accurate compared to the simple linear regression model [75]. Spatial stratification of defoliation plot-wise trends were examined by calculating the degree of spatial stratified heterogeneity using the q-statistics method [76] and the spatial autocorrelation coefficient, Moran’s I [77].

To model the impact of site factors and climate conditions on crown vitality indicators, we used linear mixed effects models (LMM) [78]. Prior to adding climate predictors, a default model was fitted:

(1) $$\begin{array}{l} {\mathrm{DEF}}_{\mathrm{it}}\;=\;{\mathrm{Year}}_{t}\;+\;{\mathrm{StandAge}}_{\mathrm{it}}\;+\;{\mathrm{Elevation}}_{i}\\ {\mathrm{SamplePlot}}_{i}\;\sim \;N(0,\;\sigma^{2}) \end{array}$$

where DEF*_it_* is the mean crown defoliation of European beech trees for sample plot *i* = 1, …, *n* and for year *t* = 1, …, 22, averaged over all trees at sample plot *i*. SamplePlot*_i_* is the random intercept, which is assumed to be normally distributed with mean 0 and variance σ^2^. To account for different number of trees on each plot, weights 1/α_it_ were introduced, where αit is the number of trees assessed at plot *i* and year *t*. Since defoliation represents an estimated percentage and due to the LMM requirements, mean defoliation values were divided by 100 before model fitting. First order autocorrelative term was introduced to account for temporal autocorrelation in the model. Seidling [46] states that the serial correlation of European beech that appears over a five-year period is not as distinct as in other species studied, which is contrary to our data. Ignoring serial correlation in model fitting leads to overestimated random effects and to the inflation of the empirical Type I error rates [79], therefore it is crucial to account for this during model fitting of defoliation data.

Due to high kurtosis, The Lambert W × F function [80] was applied to transform ΔDEF data to a normal distribution. Afterward, the same approach to the base model build up was applied as with DEF data, except that the first order autocorrelative term was left out since the data did not display serial correlation.

The final model selection process was based on diagnostic diagrams and a procedure defined by Johnson and Omland [81]. Spearman’s correlation coefficient (*rho*) was calculated between the crown vitality indicators and each quantitative environmental variable in order to obtain an overview of possible impacts. Of the potential independent variables, those that explain most of the variation of crown vitality indicators were selected with a recursive feature elimination approach (RFE) implemented within the random forest algorithm [82]. Selected variables which were linearly correlated with other variables and had a variance inflation factors VIF > 5, a commonly used threshold in detecting multicollinearity [83], were identified. The identified collinear variables with the lower value according to the Akaike Information Criteria (AIC) [84] were retained for further model development. This subset of uncorrelated environmental variables was used as predictor variables for developing the final models. All analyses were conducted in an R programming environment [85].

## Figures and Tables

**Table 1:** Estimated model parameters, standard errors, *t*-values, and *p*-values for mean defoliation LMM models with current year (DEF-I) and previous year climate variables (DEF-II). T—mean annual temperature, P—annual sum of precipitation, SPEI3—mean annual Standardized Precipitation Evapotranspiration Index calculated on a three-month time scale, lag—denotes previous year values.

		Estimate	Std. Error	*t* Value	*p* Value
**DEF-I**	Intercept	−7.924	1.591	−4.980	<0.001
	Year	0.004	8.03 × 10^−4^	4.934	<0.001
	Stand age	−4.70 × 10^−4^	3.16 × 10^−4^	−1.486	0.138
	Altitude	1.58 × 10^−4^	3.86 × 10^−5^	4.095	<0.01
	T	0.007	0.004	1.954	0.051
	P	−1.07 × 10^−5^	1.19 × 10^−5^	−0.902	0.367
	SPEI3	0.016	0.006	2.834	<0.01
**DEF-II**	Intercept	−7.542	1.571	−4.799	<0.001
	Year	0.004	7.92 × 10^−4^	4.767	<0.001
	Stand age	−3.68 × 10^−4^	3.09 × 10^−4^	−1.191	0.234
	Altitude	1.18 × 10^−4^	3.82 × 10^−5^	3.099	<0.01
	T_lag	0.005	0.003	1.553	0.121
	P_lag	9.67 × 10^−6^	1.19 × 10^−5^	0.813	0.417
	SPEI3_lag	−0.014	0.006	−2.501	<0.05

**Table 2:** Defoliation (DEF) and defoliation change (ΔDEF) model performance indices: Akaike Information Criterion (AIC); Conditional coefficient of determination (Conditional *R*^2^); Marginal coefficient of determination (Marginal *R*^2^); Intraclass Correlation Coefficient (ICC); Root Mean Square Error (RMSE).

	AIC	Conditional *R*^2^	Marginal *R*^2^	ICC	RMSE
**DEF-I**	−1906	0.89	0.60	0.71	0.061
**DEF-II**	−1915	0.88	0.62	0.68	0.062
**ΔDEF-I**	1824	0.09	0.09	1.20 × 10^−8^	0.996
**ΔDEF-II**	1806	0.32	0.32	7.07 × 10^−9^	0.984

**Table 3:** Estimated regression parameters, standard errors, *t*-values, and *p*-values for mean defoliation change LMM models with current year (ΔDEF-I) and previous year climate variables (ΔDEF-II). T—mean annual temperature, P—annual sum of precipitation, SPEI3—mean annual Standardized Precipitation Evapotranspiration Index calculated on a three-month time scale, lag—denotes previous year values.

		Estimate	Std. Error	*t* Value	*p* Value
**ΔDEF-I**	Intercept	25.114	15.641	1.606	0.109
	Year	−0.013	0.008	−1.595	0.111
	Stand age	−1.91 × 10^−4^	0.002	−0.104	0.917
	Altitude	−1.50 × 10^−4^	3.87 × 10^−4^	−0.388	0.701
	T	0.012	0.057	0.207	0.836
	P	1.47 × 10^−4^	1.35 × 10^−4^	1.094	0.274
	SPEI3	−0.006	0.091	−0.066	0.947
**ΔDEF-II**	Intercept	15.966	15.833	1.008	0.314
	Year	−0.008	0.008	−0.964	0.336
	Stand age	−2.50 × 10^−5^	0.002	−0.014	0.989
	Altitude	−0.001	3.78 × 10^−4^	−1.388	0.176
	T_lag	−0.047	0.055	−0.857	0.392
	P_lag	1.98 × 10^−4^	1.33 × 10^−4^	1.487	0.138
	SPEI3_lag	−0.388	0.089	−4.338	<0.01

## Data Availability

The data presented in this study are available on request from the corresponding author. The data are not publicly available due to legal reasons.

## Supplementary Materials

The following are available online at https://www.mdpi.com/article/10.3390/plants11060730/s1, References [86,87,88,89,90,91] are cited in the Supplementary Materials. Figure S1. Linear regression fits (black lines) and kernel smoothing functions (colour lines) for one-year smoothed mean monthly temperature, monthly precipitation sum and mean monthly SPEI data (grey lines) on the research plots during the vegetation period., Figure S2. Distribution of 28 research plots by stand age, altitude, and orientation, Table S1. Additional site factor variables and data sources, Table S2. Descriptive statistics of soil chemical properties from 28 research plots and the applied methods of analysis
