# Supplementary material for: Defoliation Change of European Beech (Fagus sylvatica L.) Depends on Previous Year Drought
Source: Plants (Basel). 2022 Mar 9;11(6):730. doi: 10.3390/plants11060730 (PMC8955490; doi:10.3390/plants11060730)
Supplement: Supplementary file 1 [file plants-11-00730-s001.zip › plants-1591214-supplementary.pdf]

Supplement material

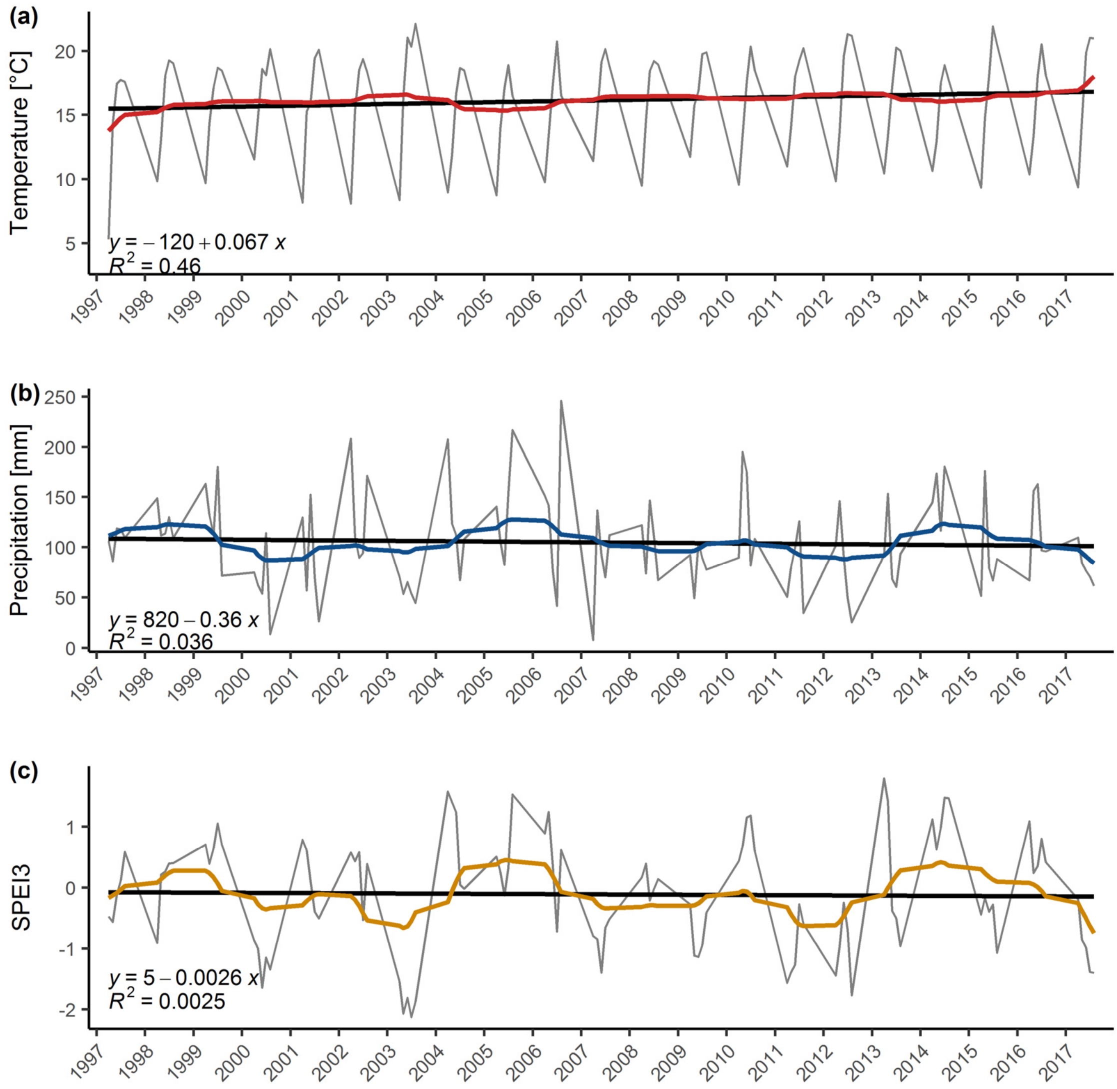

Figure S1. Linear regression fits (black lines) and kernel smoothing functions (color lines) for one-year smoothed mean monthly temperature, monthly precipitation sum and mean monthly SPEI data (grey lines) on the research plots during the vegetation period. Time series analyses [1] indicated that the temporal increase of temperature for 1997-2017 was statistically significant (p<0.001), while there was no significant trend for precipitation and SPEI.

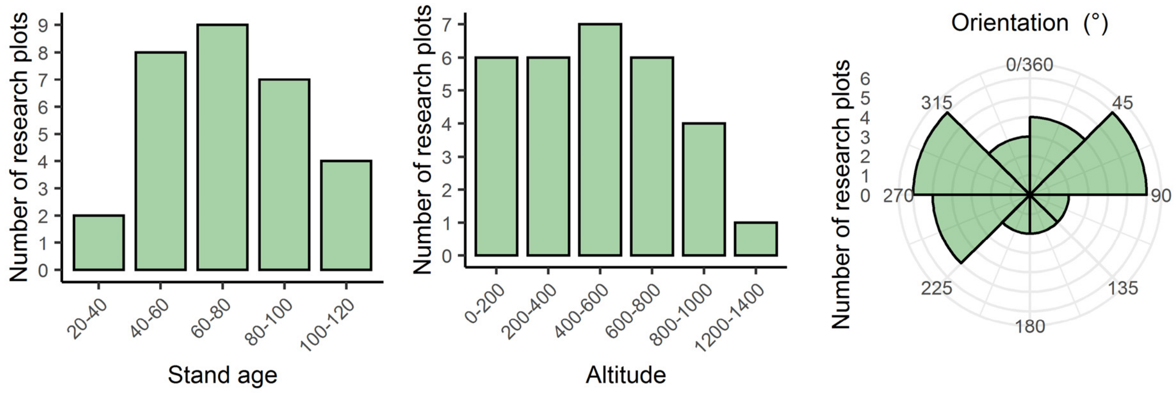

Figure S2. Distribution of 28 research plots by stand age, altitude and orientation

Table S1. Additional site factor variables and data sources

Parameter	Source
Altitude (m)	Digital Elevation Model [2]
Latitude (WGS 84)	ICP Forests database
Longitude (WGS 84)	ICP Forests database
Orientation (°)	Field measurement
Stand age	ICP Forests database

Table S2. Descriptive statistics of soil chemical properties from 28 research plots and the applied methods of analysis.

	Minimum	Maximum	Mean	Standard deviation	Method
Total nitrogen (N_{total})	0,01	0,92	0,19	0,17	Elementary analysis [3]
Available phosphorus (P)	0,12	15,32	1,72	2,19	Spectrophotometry, molybdenum blue method [4]
Available potassium (K)	4,88	36,27	9,83	5,09	AES Flame [5]
pH	3,12	7,4	4,77	1,12	Potentiometry [6]

References

1. Shumway, R.H.; Stoffer, D.S.; Stoffer, D.S. *Time series analysis and its applications*; Springer: 2000; Volume 3.
2. EEA. European Digital Elevation Model (EU-DEM), version 1.1, European Environment Agency (EEA), https://land.copernicus.eu/imagery-in-situ/eu-dem/eu-dem-v1.1?tab=metadata. **2016**.
3. ISO-13878. Soil quality - Determination of total nitrogen content by dry combustion ("elemental analysis"). **1998**.
4. Egnér, H.; Riehm, H.; Domingo, W.R. Untersuchungen über die chemische Bodenanalyse als Grundlage für die Beurteilung des Nährstoffzustandes der Böden. II. Chemische Extraktionsmethoden zur Phosphor-und Kaliumbestimmung. *Kungliga Lantbrukshögskolans Annaler* **1960**, *26*, 199-215.
5. Škorić, A. *Priručnik za pedološka istraživanja*; Fakultet poljoprivrednih znanosti: Zagreb, 1985.
6. ISO-10390. Soil quality - Determination of pH. **2005**.
